# Progress in Model Systems of Cystic Fibrosis Mucosal Inflammation to Understand Aberrant Neutrophil Activity

**DOI:** 10.3389/fimmu.2020.00595

**Published:** 2020-04-07

**Authors:** Daniel R. Laucirica, Luke W. Garratt, Anthony Kicic

**Affiliations:** ^1^Faculty of Health and Medical Sciences, University of Western Australia, Nedlands, WA, Australia; ^2^Telethon Kids Institute, University of Western Australia, Nedlands, WA, Australia; ^3^Department of Respiratory and Sleep Medicine, Perth Children's Hospital, Nedlands, WA, Australia; ^4^School of Public Health, Curtin University, Bentley, WA, Australia

**Keywords:** cystic fibrosis, neutrophil, inflammation, infection, model systems

## Abstract

In response to recurrent infection in cystic fibrosis (CF), powerful innate immune signals trigger polymorphonuclear neutrophil recruitment into the airway lumen. Exaggerated neutrophil proteolytic activity results in sustained inflammation and scarring of the airways. Consequently, neutrophils and their secretions are reliable clinical biomarkers of lung disease progression. As neutrophils are required to clear infection and yet a direct cause of airway damage, modulating adverse neutrophil activity while preserving their pathogen fighting function remains a key area of CF research. The factors that drive their pathological behavior are still under investigation, especially in early disease when aberrant neutrophil behavior first becomes evident. Here we examine the latest findings of neutrophils in pediatric CF lung disease and proposed mechanisms of their pathogenicity. Highlighted in this review are current and emerging experimental methods for assessing CF mucosal immunity and human neutrophil function in the laboratory.

## Introduction

Polymorphonuclear neutrophils are the most abundant immune cells in human blood and act as first responders to sites of infection. Their function is a key component of host defense against invading pathogens. In the autosomal recessive disorder cystic fibrosis (CF), persistent microbial colonization in the lungs induces abundant and continuous migration of neutrophils to the airways via powerful inflammatory signals of IL-6, IL-8, and leukotriene B4 (1). Recruited CF neutrophils secrete high levels of proteolytic compounds such as neutrophil elastase (NE), which damage airway tissue and highly correlate with disease severity (2, 3). Despite recognition of neutrophils in the progression of CF lung disease, mechanisms modulating their pathological role are not well-characterized. Past investigations have been hampered by a lack of widely available CF animal models and no suitable *in vitro* infection models that effectively incorporate multiple factors driving complex *in vivo* disease. New data from modern clinical studies are changing the view that neutrophils are a fixed population and are revealing a spectrum of functional phenotypes neutrophils employ to address the variety of pathogenic scenarios they encounter (4). Understanding neutrophil phenotypes and mechanisms in early CF disease, when the airway environment is less complex and more responsive to intervention, will require researchers to revisit or adapt many models of CF. With this review, we present new insights, challenges, and considerations for researchers studying neutrophils in early CF lung disease.

## Pediatric CF Lung Disease

The clinical picture of early life cystic fibrosis has changed significantly since CF was first identified in the mid twentieth century, when patients rarely survived to the age of 10 (5). Improvements in diagnostics, from sweat tests to genetic testing, and wider screening of newborns by immunoreactive trypsinogen, has permitted earlier detection of CF and management of the disease. Increased antibacterial therapy, mucolytic and osmotic agents, and the advent of CFTR modulators have further increased the lifespan of many CF patients to beyond 40 years of age (6). Despite these advances, signs of airway inflammation and lung damage are still evident in CF from an early age. In 2005, a pioneering study assessing bronchoalveolar lavage fluid (BALF) after CF newborn screening demonstrated that infection in the first year of life is linked to early airway inflammation (7). Subsequent surveillance studies have now established that for most children, inflammation, altered microbiome, active neutrophil proteolytic function, and lung damage all become evident within the first 2 years of life (8–11), before children are old enough to be treated with CFTR modulators (12, 13). Lung function declines can be evident in infants and continue into childhood (14–16). However, early airway disease can occur in the absence of overt respiratory symptoms (17) or infection (18, 19). Computed Tomography (CT) screening has revealed that CF associated structural changes diagnosed in early life persist into childhood and adolescence. Permanent bronchial wall thickening, or bronchiectasis, is detectable in about 8.5% of pediatric CF patients in the first year of life, and this increases to 36% by 4 years (8). In addition to cytokine release, neutrophil influx into early CF airways may be supported by chemotactic fragments from the extracellular matrix (20, 21). Overall, CF lung damage and declines in function are linked to neutrophil counts and levels of neutrophil proteases, that are often a response to early incidence of infection. Understanding the pathology of early lung disease as it appears today will be key to maximizing long-term benefits from subsequent CFTR modulator therapies.

## Neutrophils in CF Airways

### Neutrophil Elastase and Serine Proteases

Early in vertebrate immunity, neutrophils evolved a granule system to separately store enzymes and antimicrobial factors safely until fused with a phagosome (22). Neutrophil elastase (NE) and other serine proteases are a central component of the neutrophil antimicrobial arsenal, stored in the primary granules that are the last granule to mobilize and are highly resistant to fusion with the outer membrane (23). Yet uninhibited NE activity can be detected in over 30% of BALF samples from young children with CF (11, 24). Activity of NE is considered one of the most significant biomarkers in CF lung disease, as activity significantly correlates with lung damage and functional declines at all stages of life with CF (25–28). Unregulated activity is destructive to airway epithelial cells and the lamina propria, and can impede microbial clearance through destruction of host immune factors (29). *In vitro* and *in vivo* studies have demonstrated how elevated NE activity induces epithelial senescence in CF airway cells (30, 31), prevents epithelial repair mechanisms (24), and is a key driver of airway inflammation and mucus production (25–28). Neutrophil Elastase and other serine proteases digest a variety of host proteins, suggesting multiple mechanisms that implicate these compounds in CF airway pathology. Along with neutrophil serine proteases cathepsin G and proteinase 3, NE directly interacts with cytokines, including IL-8 and IL-1α, increasing their potency (32–34). Counter-intuitively, serine proteases also degrade antimicrobial peptides (AMPs), including lactoferrin, midkine, and surfactant protein-A (SP-A) (35–37). In particular, NE has been shown to degrade pattern recognition receptors including toll-like receptor 4 (TLR4), reducing bacterial lipopolysaccharide (LPS) sensitivity and increasing inflammation (38). In addition to modulating mucosal immunity, serine proteases may promote airway epithelial dysfunction in CF. For example, NE cleaves E-cadherin, an important component of adherens junctions, compromising epithelial integrity (39). It also induces CFTR protein degradation by calpain activity in both *in vitro* epithelial cells and *in vivo* mouse models, resulting in impaired channel function as well as increasing sodium transport into cells through proteolytic activation of sodium ion channels (ENaC) (40–42).

### Cysteine Proteases, Matrix Metalloproteinases, and Reactive Oxygen Species

In addition to NE and other enzymes found in primary granules, additional neutrophil derived compounds may contribute to CF airway pathology. Crucial for intracellular degradation of pathogens, secreted cysteine proteases have similar deleterious effects as their serine counterparts. Cathepsins B and S positively correlate with clinical markers of inflammation in pediatric CF airways, including NE, IL-8, and TNFα (43, 44). They selectively maintain neutrophil influx through activation of chemokines containing glutamic acid-leucine-arginine (ELR) motifs and inactivation of lymphocyte attracting non-ELR chemokines (45). Similar to serine proteases, cathepsins can compromise immunity through degradation of AMPs such as lactoferrin, LL-37, SP-A, and β-defensins (46–49). Cathepsins B and S are also implicated in airway mucus dehydration through induction of ENaC activity (50, 51). Matrix metalloproteinases (MMPs) are additional proteases implicated in CF associated with airway remodeling following lung injury. They can originate from any tissue, but neutrophil derived MMP-9 is particularly linked to airway damage, inflammation, and lung function decline in early CF (52, 53). Furthermore, MMP-9 sustains airway neutrophilia through potentiation of IL-8 and generation of proline-glycine-proline (PGP) matrikine fragments from breakdown of collagen (21, 54). Upon phagocytosis of pathogens, neutrophils produce large amounts of superoxide radicals for microbial killing. Broadly termed reactive oxygen species (ROS), neutrophils are among the most potent producers of these compounds (55). Oxidative stress as shown by elevated airway ROS is observed in chronic obstructive pulmonary disease (COPD) as well as CF (56–58). Increased ROS production results in destruction of antiproteases, which are crucial for protecting tissue from unregulated proteolysis (59, 60). In the context of CF, ROS may impede the function of antiproteases such as alpha-1-antitrypsin, an important NE inhibitor, prolonging airway neutrophil proteolytic activity (24, 61).

### CFTR in Neutrophils

A central conundrum of CF is why proteolytic activity develops in such early, mild stages of CF lung inflammation. One obvious area of investigation has been whether neutrophil dysfunction in CF airways is exclusively influenced by factors in the lung environment or is also a consequence of inherent CFTR defects. Since the discovery of the CFTR gene, there have been studies suggesting CFTR protein is routinely expressed in cells of myeloid origin and has a role in microbial clearance within phagosomes (62–64). Hypochlorous acid (HOCl) is an important antimicrobial component of neutrophil phagosomes whose formation is proposed to be dependent on CFTR-mediated chloride ion transport (65). CFTR is reported to traffic to phagosomal membranes in peripheral blood neutrophils, with CFTR mutation resulting in defective phagosomal chlorination, affecting clearance of microbes such as *P. aeruginosa* (66–68). Contrasting findings have shown normal respiratory burst activity and production of nicotinamide adenine dinucleotide phosphate (NADPH) oxidase components in CF blood neutrophils and no detectable CFTR protein in these cells (69). Additional evidence for the role of CFTR in neutrophils comes from a small number of studies showing restoration of CF neutrophil functions including CFTR phagosomal trafficking (67), leukocyte activation (70), and intracellular ion regulation (71) in response to CFTR modulator treatment. Still, further research is needed to clarify the presence and function of CFTR in neutrophils, and how defects in the gene influence the pathological activity of CF airway neutrophils. One consistent observation is a CFTR mutation dependent effect on *in vivo* neutrophil lifespan, with CF neutrophils displaying delayed apoptosis compared to non-CF neutrophils, possibly preventing resolution of neutrophilic inflammation (72–74). The most recent of these studies demonstrated a link between delayed apoptosis by CF neutrophils and propensity to form neutrophil extracellular traps (74).

### Neutrophil Extracellular Traps

The identification of neutrophil extracellular traps (NETs), extracellular networks of DNA containing azurophilic granules, neutrophil elastase and other antimicrobial components, was a significant event in neutrophil biology (75). NET formation was initially viewed as a form of active cell death upon which nuclear and granular membranes were disintegrated, contents ejected and mixed in the cytoplasm, then released upon deterioration of the cell membrane (76). The process was later termed NETosis and proposed to be an alternative strategy used by neutrophils upon failing to clear infection via traditional phagocytosis. While NETs can trap and neutralize invading pathogens, the extent of their microbe killing abilities is debated (77, 78). A significant amount of research into NETosis has been undertaken, as recently reviewed by this journal (79). Multiple studies have described forms of NETosis that result in mitochondrial DNA release rather than nuclear DNA, or allow neutrophils to remain viable and motile after NET formation (80–83). The ability of NETs to harbor NE, the presence of NET derived DNA in CF sputum, and increased pathogen resistance in response to NETs, suggest NETosis is likely to play a role in CF lung disease (84, 85). Yet the question remains on how frequently NETosis occurs during early CF airway inflammation, prior to significant biofilm formation that reduces availability of bacteria to neutrophils.

### Neutrophil Exocytosis

Perhaps the most intriguing hypothesis explaining early airway neutrophil proteolytic activity is that upon recruitment to CF airways, neutrophils reprogram toward an aberrant granular-releasing, immunoregulatory, and metabolically distinct (GRIM) phenotype that includes exocytosis of primary granules—as evidenced by high CD63 expression (86–88). The GRIM phenotype is specific to recruited neutrophils as peripheral blood neutrophils from CF patients exhibit a normal phenotype (89). However, when naïve neutrophils from either CF or non-CF donors are stimulated in an *in vitro* transmigration model of neutrophil recruitment by adult CF BALF or sputum, cells from both groups of donors undergo GRIM reprogramming (89). While factors such as tumor necrosis factor-alpha (TNF-α) can prime exocytosis of neutrophil azurophilic granules (90), Forrest and colleagues observed GRIM reprogramming only upon stimulation with *ex vivo* CF samples but not with exogenously added chemokines, suggesting a yet unidentified factor in CF airways is responsible for changes in neutrophil activity (89).

Most significantly, GRIM neutrophils were also found to have reduced bacterial killing capacity, which aligns with the apparent disconnect between NE release and inability to resolve infection in CF airways (89). More recent studies have reported how Staphylococcal superantigen-like protein 13 (SSL13) from *Staphylococcus aureus*, a common early CF pathogen, can induce neutrophil exocytosis (91) and whose production is evident in the CF microbiome (92). In an age related cohort of non-CF children admitted for acute respiratory distress syndrome (ARDS), neutrophil exocytosis and reduced bacterial killing was observed in individuals co-infected with virus and bacteria but not viral infection alone, suggesting that neutrophil exocytosis may be linked to responses against polymicrobial infection (93). This relationship with infection is yet to be established in early CF disease, however neutrophil exocytosis markers correlate positively with disease severity more so than free NE activity (11). Therefore, changes in airway neutrophil functional markers may be more reliable indicators of disease progression in children with CF and should be a focus of early CF lung disease research.

## Modeling Infection And Inflammation

**Figure 1:**
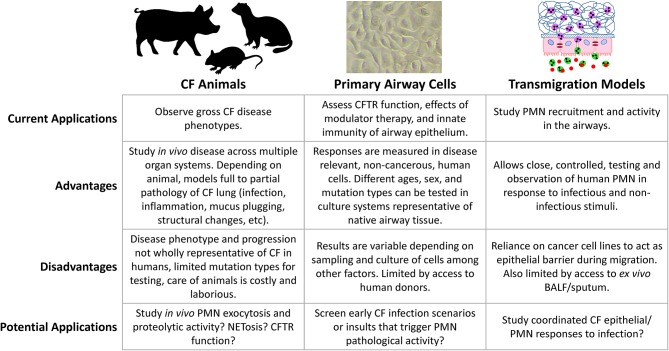
Comparison of model systems for studying CF lung disease.

Characterizing the early mechanisms that trigger phenotype shifts in airway neutrophils may be key for preventing progressive lung disease. Clinical surveillance gives valuable insights into disease phenotypes *in vivo*, however, basic science is crucial for understanding the biology of CF lung disease and the role of the airway epithelium. Over the years, researchers have developed a variety of approaches for this purpose (Figure 1). The following is a summary of some of the more important, biologically relevant models currently in use to study infection and inflammation in CF airways.

### Animal Models

While CFTR mutant and knockout mice were developed shortly after discovery of the CFTR gene, their use as animal models for CF lung disease is controversial as they lack a robust CF lung phenotype of spontaneous infection and disease (94–97). Despite extensive similarity, mouse immune cells can behave differently to human counterparts in their response to pathogens, for example, murine neutrophils are not activated by SSL13 (91). Mice also express toll-like receptor 11 (TLR11), a TLR not expressed in humans, that detects profilin and bacterial flagellin (98). Still, mouse models of induced airway infection have provided insights into CF airway inflammation and disease. Studies of acute *Pseudomonas aeruginosa* infection have observed poor growth, increased mortality, and reduced bacterial clearance in CF vs. wild-type mice (99, 100). Additionally, CF mice have exaggerated levels of murine inflammatory cytokines and airway neutrophilia in response to infection, as well as prolonged inflammation compared to wild-type mice (101, 102). Most of these studies have inoculated animals through intratracheal delivery of agarose beads embedded with bacteria, an unrealistic representation of how CF patients normally acquire these organisms. Of interest has been the observation that environmental acquisition of *P. aeruginosa* can be modeled in mice through inoculated drinking water, with CF mice more susceptible to chronic colonization via this route (103). Chronic exposure of CF mice to *P. aeruginosa* LPS also results in increased airway inflammation, neutrophilia and airway remodeling (104, 105). A common theme emerging from these studies, is that neutrophils and their products play a central role in CF lung pathology. With the development of Cre recombinase mice targeting the neutrophil-specific locus Ly6G (106), future studies utilizing this model will continue to play a very useful role in elucidating CF airway neutrophil biology.

The more physiologically relevant animal model for studying CF lung disease include CFTR disrupted pigs and ferrets, as they recapitulate the CF phenotype across all organ systems implicated in human disease (107). Both models were developed just over a decade ago using adenoviral vectors, generating CFTR full or partial knockout animals in both species via exon 10 disruption, as well as a ΔF508 pig (108–110). Pigs are suitable human disease models due to their analogous physiology, and in the case of respiratory disease, similar bronchial structure and distribution of submucosal glands (111). CF pigs have CFTR protein similar to that of humans (112). Neonatal CF pigs have little airway inflammation and normal levels of IL-8 and neutrophil counts in BAL compared to non-CF pigs (109). Neonatal CF pigs also have increased presence of microbes in the lungs as shown by culture from *ex vivo* tissue samples, and are less likely to have sterile BAL samples compared to non-CF pigs (113). In the months following birth, CF pigs develop signs of lung disease such as mucus accumulation, inflammation, infection, and airway remodeling (113). While lung disease progression in the CF pig model reflects progression in humans, there are obvious drawbacks of cost and time of pig husbandry and the need of adequate facilities and resources. Furthermore, virtually all CF pigs develop meconium ileus and require early surgical intervention; in contrast, the condition is present in only 20% of infants with CF (109, 113, 114).

Ferret CFTR protein length, amino acid sequence, and function is also similar to that of humans (115). Like CF humans, CF ferrets are prone to spontaneous airway infection; however, infection in these animals is far more severe, with CF ferrets requiring continuous antibiotic treatment immediately after birth to survive (116). Additionally, CF ferrets demonstrate abnormally high levels of lung inflammation from birth, and lung disease progresses rapidly upon cessation of prophylactic antibiotics (117, 118). As a result, CF ferrets may not be an ideal system to model the slow progressive lung disease observed in humans, as their disease phenotype develops too quickly. However, a recent study developed homozygous CFTR^G551D/G551D^ ferrets to test effects of *in utero* treatment with VX-770 (ivacaftor) (119). Prenatal and early postnatal administration ameliorated CF multi-organ disease, posing new research questions around CFTR in early development, the possibility of prenatal modulator therapy, and disease attenuation in CF animals to further study effects of modulator treatment or model mild disease in humans. While neutrophil counts and elastase activity in CF animals trend similarly to human disease, neutrophil reprogramming has yet to be evaluated. Future studies must assess airway neutrophil exocytosis and lung disease severity in CF animals to determine if they are suitable models for characterizing this process in humans.

### Primary Airway Epithelial Cells

While animal models allow observation of gross phenotype of disease, *in vitro* studies permit experimentation in a highly controlled environment and are important for understanding mechanisms of disease at the cellular level. The accepted gold standard for *in vitro* CF research are patient derived primary airway epithelial cells (pAEC). As a barrier that protects the lung from direct environmental exposure, the airway epithelium has long been recognized for its role in host defense and respiratory disease (120–125). Cells are typically isolated from epithelial brushings of the nose or lower airways, or less frequently from explanted lungs (126, 127). Yields from brushings are variable and *ex vivo* pAEC have limited proliferative capacity; they become senescent after only a few passages making them difficult to expand in culture (126, 127). The adaptation of conditionally reprogrammed airway epithelial cells (CRAEC) through co-culture with irradiated fibroblast feeder cells has significantly increased passage number capacity of pAEC, while maintaining lineage specific characteristics (128). Additionally, CRAEC can be seeded from co-culture into air-liquid interface culture (ALI) to form a differentiated pseudostratified epithelial layer (128, 129). This has enabled many CF research groups to look to CF primary airway epithelial cell models in order to understand the cellular drivers of progressive lung disease, and more recently to evaluate the efficacy of CFTR modulators in restoring CFTR function in target cells (130, 131). Nasal pAEC are increasingly being used in epithelial CFTR studies, since their growth, differentiation, CFTR activity, and response to modulators are similar to lower airway cells, and have the advantage of being more readily accessible (129). Nasal cells have also been adapted to three dimensional spheroid cultures that are representative of native epithelium and mature more quickly than traditional ALI cultures (132). These spheroids have then been used to quantify CFTR function via spheroid swelling in cultures from CF patients across different mutation classes, to assess individual responses to modulator treatment (133, 134). As such, they have potential as a preclinical screening tool to identify responses to modulator therapies in a personalized medicine approach.

Despite increased airway inflammation in CF patients, there is still debate as to whether the CF airway epithelium is inherently pro-inflammatory (135–137). Baseline expression of neutrophil chemoattractants including IL-8, IL-6, and IL-1β is reported in some studies to be similar in CF vs. non-CF pAEC (138–140), but others report increases in CF cells at baseline (141, 142). Increased airway inflammation could also be a result of dysfunctional CF epithelial innate immunity, a major topic in CF research, as the airway epithelium has an important role in responding to infection and neutrophil recruitment (143–147). Studies have shown IL-8 release and NF-κB activity are increased in CF vs. non-CF pAEC following *P. aeruginosa* infection (142, 148, 149). CF pAEC have also been shown to display differential gene expression at the transcriptional level compared to non-CF pAEC in response to *P. aeruginosa* infection, which may be further evidence of CF aberrant immune responses (150, 151). In response to infection with respiratory viruses, studies have also observed increased IL-8 production in CF vs. non-CF pAEC (152, 153), which is analogous to *in vivo* findings in pediatric CF patients with rhinovirus infection (154). However, other studies have reported no difference in inflammatory cytokine production as a result of *in vitro* viral infection (155, 156). The filamentous fungi *Aspergillus fumigatus* is emerging as an important early life CF pathogen increasingly detected in pediatric CF airways (157, 158), with *A. fumigatus* infection associated with increased air trapping among 5 year old CF patients (159). Two *in vitro* studies to date have used immortalized cell lines and reported altered cytokine production, though specific data were conflicting (160, 161). Assessing innate immune responses to fungal pathogens in CF will be key to determining treatment priority, but innate immune mechanisms have yet to be corroborated in CF pAEC.

Variability amongst findings from pAEC infection studies could be attributed to the originating cohorts, sampling differences, age and disease severity of subjects, culture methods, and use of differentiated vs. undifferentiated cultures. Additionally, the selection of pathogens can affect outcomes, as pAEC responses can be heterogeneous to individual strains or isolates of the same species (162). Primary cells will continue to be important tools for addressing unanswered questions in CF lung disease, including how epithelial immunity is linked to neutrophil inflammation, how the epithelium responds to fungal and polymicrobial infection, how the airway microbiome affects epithelial homeostasis, and whether CFTR modulators have effects on airway innate immune signaling. Researchers must think carefully about the above factors and how they influence experimental outcomes in pAEC, especially if findings are translated to lung disease pathogenesis in CF patients.

### Neutrophil Transmigration to the Airways

The epithelium is not only a barrier to external pathogens, but also presents an obstacle to responding neutrophils. *In vitro* replication of this mucosal physiology was established by early studies assessing neutrophil transmigration across the intestinal mucosa (163–166). These studies established polarized epithelial cultures onto inverted permeable inserts, which were turned over prior to migration for direct loading of naïve isolated neutrophils and thus model basolateral to apical neutrophil migration. It has since been adapted to characterize transmigration across lung epithelium (167), assess the role of neutrophils in β-catenin mediated airway epithelial repair (168, 169), as well as describe responses to infection with respiratory syncytial virus (RSV) and *P. aeruginosa* (170–172). The model previously mentioned in this review uses Alvetex™ 3D scaffolds rather than permeable membranes, which better replicate neutrophil swarming (89, 93), to study neutrophil responses to CF sputum. To understand factors driving early neutrophil fate including exocytosis, this same model could be applied with pediatric BALF. However, pediatric *ex vivo* samples are difficult to obtain, often of limited volume compared to samples from adults. One approach yet to be fully utilized is to apply material from infected CF pAEC as surrogates for human samples. This has multiple benefits. Robust models of pAEC infection responses are well-established and because pAEC can be bio-banked for downstream culture and infection, material can be generated as needed. This approach also facilitates a more focused assessment of factors influencing neutrophil functions, such as epithelial responses to specific infection scenarios.

One caveat of past transmigration studies is the dependence on lung cancer derived cells lines, such as A549, H292, H441, 16HBE, and Calu-3 cells, as a substitute for primary cell derived epithelium. Advantages include easy access to cell lines and robust growth in culture, but at the cost of interpreting epithelial responses during migration through cancer cells. This is critical, since neutrophils directly change airway epithelium via microRNA (173) or exosomes (174) and neoplastic cells may not reproduce CF pAEC responses. As limitations on pAEC culture expansion are overcome, transmigration studies are increasingly incorporating pAEC (175). A remaining challenge is that established methods for differentiating pAEC traditionally employ 0.4 μm pore size inserts, but a 3.0μm pore size or larger is required to permit neutrophil transmigration, which can result in significant loss of primary cells during seeding. One study has managed to address this issue by coating both faces of a transwell insert with extracellular matrix and providing seeded cells with laminins to improve attachment (175). Primary cells differentiated into pseudostratified epithelial layers on a 3.0 μm insert, similar to how they would on a conventional 0.4 μm transwell insert, and permitted neutrophil transmigration upon apical infection with *P. aeruginosa* (175). Future integration of CF pAEC in models of neutrophil transmigration will be required for studying coordinated immune responses of the CF airway epithelium and recruited neutrophils in a single translational system. If designed with high throughput screening in mind, there is great potential to facilitate much needed pre-clinical testing of anti-inflammatory drugs in CF.

## CFTR Modulator Therapy And Airway Inflammation

Depending on their mechanism of action, CFTR modulators are characterized as correctors that improve defective CFTR trafficking to the cell surface, or potentiators that enhance defective CFTR function. Studies of modulators have shown improvements in patients as measured by sweat chloride levels and FEV_1_, however, efficacy against infection and airway inflammation is poorly investigated. In placebo controlled studies of lumacaftor-ivacaftor and tezacaftor-ivacaftor in CF patients ≥12 years of age, infective pulmonary exacerbations occurred at similar rates in both treatment and placebo groups (176–178). Phase 3 trials of lumacaftor-ivacaftor in CF patients aged 6–11 also found that incidence of infection associated pulmonary exacerbations was similar between patients receiving treatment (18%) and patients receiving a placebo control (19%) (179). The recently FDA approved elexacaftor-tezacaftor-ivacaftor triple therapy roughly halves the incidence of infective pulmonary excacerbations compared to a placebo (180), but the drug is not yet approved for patients under 12 years of age. Multiple studies have shown that administration of modulators reduces bacterial colonization within the first year of treatment and delays acquisition in uncolonized patients; however, bacterial isolates present in the airways prior to treatment persist and may eventually rebound over longer periods (12, 13, 181, 182). Whether CFTR modulators reduce levels of inflammatory cytokines is still not certain, as there is evidence of both reduction and no effect on clinically relevant biomarkers including NE (12, 13). Altogether, current findings suggest that modulator therapy alone may not be sufficient to manage infection and airway inflammation in this population, especially over the long term.

## Conclusion

**Figure 2:**
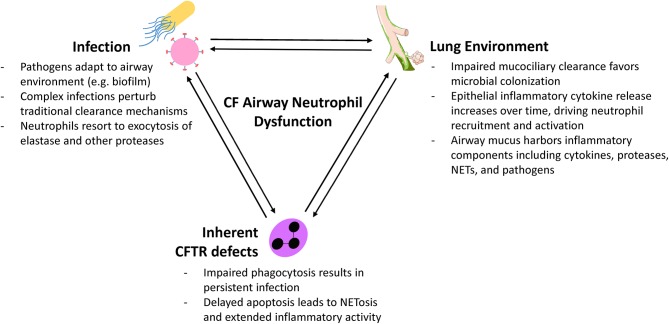
Factors influencing CF airway neutrophil plasticity.

Neutrophils have a major role in CF lung disease but our ability to treat the underlying mechanisms is still limited. Modern approaches are revealing new perspectives on neutrophils as plastic, programmable drivers of airway disease who both respond to and actively shape the local airway environment (Figure 2). These novel neutrophil functions are occurring even in mild and largely asymptomatic pediatric CF lung disease and precede structural lung changes. Even with the advent of combination CFTR modulator therapy, which improves lung function but perhaps not infection and inflammation, continued investigation of initial neutrophil pathological activity is necessary to identify much-needed interventions that can address this problem. Researchers now have available a diverse number of tools for understanding the complex interplay between infection, the airway epithelium, and recruited neutrophils (Figure 1). Moving forward, basic studies will need to consider the advantages of various approaches, caveats, and carefully select appropriate models when exploring the beginnings of CF airway neutrophilic disease.

## Author Contributions

DL, LG, and AK conceived the review. DL and LG conducted literature review and wrote the manuscript. LG and AK provided critical review.

### Conflict of Interest

LG and AK are co-investigators with some of the authors cited within the review. The remaining author declares that the research was conducted in the absence of any commercial or financial relationships that could be construed as a potential conflict of interest.
